# Potential Anti-Inflammatory and Anti-Fatigue Effects of an Oral Food Supplement in Long COVID Patients

**DOI:** 10.3390/ph17040463

**Published:** 2024-04-05

**Authors:** Annalisa Noce, Giulia Marrone, Manuela Di Lauro, Chiara Vita, Giulia Montalto, Gloria Giorgino, Carlo Chiaramonte, Cartesio D’Agostini, Sergio Bernardini, Massimo Pieri

**Affiliations:** 1 UOSD Nephrology and Dialysis, Department of Systems Medicine, University of Rome Tor Vergata, 00133 Rome, Italy; dilauromanuela@gmail.com; 2 QuMAP-PIN, University Center “Città di Prato” Educational and Scientific Services for the University of Florence, 59100 Prato, Italy; 3 School of Specialization in Nephrology, University of Rome Tor Vergata, 00133 Rome, Italy; 4 Department of Statistics, University of Rome Tor Vergata, 00133 Rome, Italy; 5 Department of Experimental Medicine, University of Rome Tor Vergata, 00133 Rome, Italy; 6 Laboratory of Clinical Microbiology, Policlinico Tor Vergata, 00133 Rome, Italy; 7 Department of Laboratory Medicine, Tor Vergata University Hospital, 00133 Rome, Italy

**Keywords:** SARS-CoV-2, cytokine storm, oxidative stress, fatigue, long COVID syndrome, adjuvant treatment, natural bioactive compounds, inflammatory state, quality of life

## Abstract

Long coronavirus disease (COVID) syndrome leads to chronic inflammatory state onset that can have a multisystem impact and compromise organ function. Moreover, long COVID syndrome is often characterized by the presence of chronic fatigue, which affects subjects’ daily activities and worsens their quality of life. The aim of our double-blind, placebo-controlled randomized trial (protocol code RS 150.21, approved on 4 November 2021) was to evaluate the beneficial effects of the consumption of 2 cps/day, for two months, of an oral food supplement (OFS), based on *Echinacea angustifolia*, rosehip, propolis, royal jelly and zinc, in long COVID patients, compared to a two-month placebo period. The OFS’s vitamin C content was equal to 22.17 mg/g (8.87 mg/capsule). The OFS’s total polyphenol content was 43.98 mg/g gallic acid equivalents. At the end of the in vivo study, we highlighted a significant decrease in the inflammatory parameters in the OFS period, compared to the placebo period (neutrophil-to-lymphocyte ratio, *p* = 0.0455; monocyte to-lymphocyte ratio, *p* = 0.0005; C-reactive protein, *p* = 0.0145). Our study also highlighted a significant increase in vitamin D serum values (*p* = 0.0005) and, at the same time, an improvement in patients’ life quality and a reduction in fatigue, monitored by the fatigue severity scale. This study showed the OFS’s beneficial effects on the inflammatory state, fatigue and quality of life in long COVID patients.

## 1. Introduction

Severe Acute Respiratory Syndrome CoronaVirus 2 (SARS-CoV-2) is a single-stranded positive RNA virus that contains four main types of proteins: nucleocapsid, membrane, envelope and spike proteins. Spike proteins, the main virulence factor in SARS-CoV-2, are a key element for viral attack on target cells. Blood chemistry in SARS-CoV-2 patients generally shows a plasma increase of pro-inflammatory cytokines, as well as changes in the leukocyte formula, of which 70% are neutrophils. In addition, serum-increased levels of C-reactive protein (CRP), erythrocyte sedimentation rate (ESR) and D-dimer in blood were also observed in SARS-CoV-2 patients [1].

The mechanisms behind the various manifestations of the infection are not fully known, but most literature data suggest that there is an immune system dysregulation caused by the virus, resulting in tissue damages [2].

In fact, SARS-CoV-2 encounters a state of acute inflammation which could “become chronic”, and immune dysregulation. In detail, a massive release of inflammatory molecules such as cytokines and chemokines were observed, a phenomenon known as “cytokine storm”, characteristic of the coronavirus disease (COVID)-19 pathogenesis [3].

Emerging data suggest that most of the clinical pictures of COVID-19 may result from a compromised immune response with a delayed viral clearance. The lack of viral clearance in the upper airways allows the entry of the virus into the lower airways and determines an acute dysfunctional response. The latter is characterized by a synergistic effect, given by the combination of a decreased viral clearance and an innate excessive immune response [4].

In addition to the clinical manifestations that characterize the acute phase of the disease, the possible symptoms that follow the SARS-CoV-2 infection can be considered, including the multisystem complications that define the long COVID syndrome (long COVID) [5].

Therefore, it can be stated that aberrant inflammatory responses and immune dysregulation persist and, similar to the acute phase of COVID-19, the adhesion of hyperactivated neutrophils to the capillaries in the lungs and in the other organs may contribute to the onset of systemic symptoms. For this reason, the effectiveness of the COVID-19 vaccine was examined in patients with different clinical conditions. Consequently, its positive impact on their immune system was demonstrated [6]. Moreover, the clinical picture refers to a multisystemic symptomatology should be also present in long COVID [7].

According to the above-mentioned evidence, in COVID-19 infection and in long COVID, the chronic systemic inflammation causes multi-organ damage and can worsen pre-existing clinical conditions, among them depressive and anxiety disorders [8].

Moreover, long COVID is often characterized by the presence of chronic fatigue syndrome (CFS) [9]. CFS is commonly characterized by severe fatigue, cognitive dysfunction and sleep disorders that severely impair the activities of daily life. The symptom of fatigue, in fact, negatively impacts the patient’s work performance, family life and social relationships. CFS patients report asthenia, brain fog, muscle weakness, delayed recovery after physical exercise and non-restorative sleep. Fatigue, in fact, is closely correlated with sleep disorders, which can be both a cause and/or a consequence of chronic fatigue [10,11]. There is a strong correlation between CFS and long COVID. In fact, it is estimated that about half of long COVID patients show the same symptoms as CFS. Several studies have found similar cognitive abnormalities and symptoms in both CFS and long COVID patients [12].

Currently, the focus of researchers is to find effective and natural treatments, characterized by few side effects, useful for counteracting systemic inflammation and fatigue in long COVID patients [13]. In the literature, several studies have shown that natural bioactive compounds (NBCs), in addition to pharmacological therapy and a healthy lifestyle, are essential for slowing down the progression of several chronic degenerative non-communicable diseases, characterized by a chronic inflammatory state and for counteracting their related complications [14,15]. In particular, in nonalcoholic fatty liver disease (NAFLD) patients, omega-3 and curcumin have a proven effect in improving the lipid metabolism [16].

In this sense, NBCs, widely used in the pharmaceutical industry for their effectiveness and absence of side effects, have been identified for their potential beneficial effects in infectious and inflammatory diseases and, therefore, were tested as a possible adjuvant therapy in the treatment of COVID-19 and long COVID patients, both in single administration and in association with standard pharmacological treatments [17].

Several previous studies [14,18,19] have shown a significant decrease in traditional inflammatory biomarkers (such as CRP, ESR, platelet/lymphocyte ratio) and in oxidative stress parameters, in different pathological conditions after the NBC assumption.

In fact, NBCs, such as *Echinacea angustifolia*, a plant commonly used to prevent upper respiratory infections, are known for their antimicrobial and anti-inflammatory properties. It has been reported that *Echinacea angustifolia* exerts a broad-spectrum antibacterial activity and is also known for its potential antiviral activity against coronavirus; in fact, a study has shown its effectiveness as an antagonist of the viral protein of SARS-CoV-2 [20]. Moreover, *Echinacea angustifolia* seems to have strong therapeutic potential, inhibiting the protein Mpro, essential for viral replication. Major constituents of *Echinacea angustifolia* roots are alkylamides, caffeic acid esters, particularly echinacoside and polysaccharides. Alkylamides have inhibitory effects on cyclooxygenase (COX) and 5-lipoxygenase and they determine the plant’s anti-inflammatory activity through the inhibition of the production of prostaglandins and leukotrienes [21]. In addition, they are capable of increasing phagocytosis, as the echinacoside does [22]. Polysaccharides can stimulate the non-specific immune response, and they appear to have a moderate effect on B lymphocytes but no activity on T lymphocytes. Among polysaccharides, heteroxylian can activate phagocytosis and anabinogalactan seems capable of inducing the production of cytokines such as IL-1, TNF-α and interferon beta-2 by macrophages. Macrophages, activated by arabinogalactan, have demonstrated cytotoxic activity against several microorganism and *versus* tumor cells [23]. *Echinacea angustifolia* has well-known immunomodulatory effects, which boost viral clearance not only in SARS-CoV-2 infection but also in human papilloma virus (HPV) infection. Echinacea extract supplementation in women with L-SIL/cervical intraepithelial neoplasia (CIN-1) significantly induces HPV lesion clearance, improving vaginal, colposcopic and histological parameters [24]. Moreover, *Echinacea angustifolia* dry root extracts seem to be a valid adjuvant therapy in reducing relapse incidence in patients treated for genital condylomatosis [25].

Rosehip contains several biologically active compounds and among these are the polyphenols. The latter are known for their antioxidant and anticancer effects. In fact, polyphenols are antioxidant substances and protective agents against chronic disease onset [26]. Extracts of rosehip fruits have shown powerful anti-inflammatory and anti-nociceptive activities [27], while rosehip powder extracts may inhibit both COX-1 and -2 [28]. In a clinical trial, it was shown that rosehip powder may be effective in hip and knee osteoarthritis patients [29]. Rosehip powder preparations also exert anti-inflammatory properties in healthy subjects, inhibiting the chemotaxis of peripheral blood neutrophils and monocytes. Moreover, these preparations may induce a decrease in CRP after 4 weeks of supplementation in patients with osteoarthritis [30,31].

Propolis is a resinous product that bees collect from the exudates of plants. Antibacterial, anti-inflammatory, antiviral, antioxidant, antifungal, antiseptic and immuno-modulatory activities are just some of its countless properties [32]. Several mechanisms are involved in its anti-inflammatory effects, including the reduction in prostaglandin biosynthesis and of inflammatory cytokines and the inhibition of COX and the nitric oxide synthesis. Propolis also has immune modulatory effects, because it decreases the expression of many pro-inflammatory genes like Adm, Il1b, Icam1, Wnt3a, Akt1, Txn1, Cdkn1b, Herpud1, Noxa1, Car9, Hes1, Hes5, Wnt5a, Vegfa and Mapk1, while upregulating the expression of other anti-inflammatory genes such as Calm1, Cav1, Dab2, Rb1, Socs3, Tnf and Wnt6. Moreover, propolis decreases the migration of immune cells, including neutrophils and macrophages, by downregulating CXCL9 and CXCL10 chemokines [33].

Royal jelly is a substance secreted by the hypopharyngeal and mandibular glands of bees and represents an important nutraceutical, a functional food as well as an oral food supplement (OFS) [34]. A bioactive substance of royal jelly called 10-hydroxy-2decenoic acid (10-HDA) plays a major role in several biological processes, including inflammation and oxidative stress [35]. It activates TRPA1 (transient receptor potential Ankyrin 1) and TRPV1 (vanilloid1) which, in turn, induce thermogenesis and increase the energy expenditure [36]. Moreover, royal jelly supplementation enlarges peroxisome proliferator-activated-alpha (PPAR-a) expression, contributing to a body weight decrease due to enhancing lipolysis [37]. Therefore, royal jelly, in addition to having an anti-inflammatory effect, shows significant action on the basal metabolism [38].

Zinc acts as an immune response modulator based on its bioavailability. A previous study [39] showed that zinc levels are inversely related to the concentration of pro-inflammatory cytokines, such as interleukin (IL)-6, IL-8 and tumor necrosis factor (TNF)-α. Therefore, experimental studies have demonstrated that the supplementation of zinc-based foods lead to a substantial reduction in inflammatory biomarkers, and this corroborates the hypothesis of its anti-inflammatory action [40].

The aim of our double-blind, placebo-controlled randomized trial was to evaluate the possible beneficial effects of the consumption of 2 cps/day of an OFS, based on *Echinacea angustifolia*, rosehip, propolis, royal jelly and zinc, in long COVID patients, for 2 months, compared to a 2-month placebo period. In particular, we evaluated its possible impact on the systemic inflammatory state, on fatigue and on life quality.

## 2. Results

The results obtained in the laboratory parameters after 2 months of OFS adoption and after 2 months of placebo adoption are shown in Table 1. At the end of the study, we highlighted a statistically significant increase in vitamin D serum values (27.96 ± 10.25 mg/dL vs. 34.54 ± 12.53 mg/dL) only after OFS adoption.

The impact of OFS on the immune system and on the inflammatory state has been evaluated (data are reported in Table 2). It is interesting to note that in our patients, a reduction in the inflammatory biomarkers was observed. In particular, the CRP decreased significantly (2.77 ± 2.5 mg/dL vs. 1.44 ± 1.70 mg/dL) after OFS treatment, as shown in Table 2 which summarizes the inflammatory parameters CRP and ESR, monitored before (T0) and after (T1) the treatment with OFS and before (T0) and after (T1) the placebo adoption.

Moreover, Table 2 shows the new inflammatory indices (neutrophil/lymphocyte ratio (NLR), platelet/lymphocyte ratio (PLR), monocyte/lymphocyte ratio (MLR) and lymphocyte/monocyte ratio (MRL)), monitored before (T0) and after (T1) the treatment with OFS and before (T0) and after (T1) the placebo adoption. A statistically significant reduction was also observed in the NLR (2.08 ± 0.81 vs. 1.77 ± 1.03) and in the MLR (0.26 ± 0.14 vs. 0.13 ± 0.05) after the OFS treatment.

Table 3 shows the parameters related to the body composition, monitored before (T0) and after (T1) the OFS treatment and before (T0) and after (T1) the placebo adoption. All the parameters examined did not change in a statistically significant manner.

Blood pressure values did not show any statistically significant changes after the treatments, as reported in Table 4.

**Figure 1:**
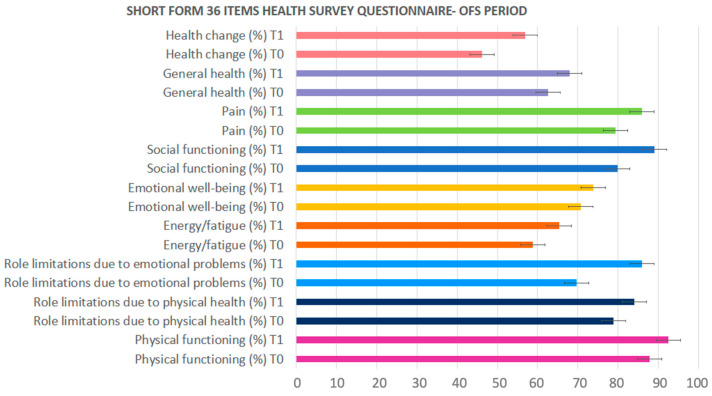
Nine functional topics of the SF-36 questionnaire in the oral-food-supplement-treated patients. Values are expressed as percentages.

**Figure 2:**
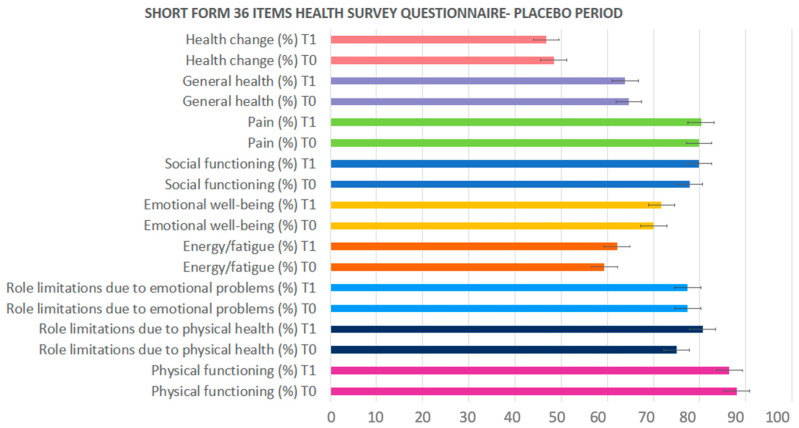
Nine functional topics of the SF-36 questionnaire in the placebo-treated patients. Values are expressed as percentages.

Figure 1 and Figure 2 show the percentage values of the nine functional domains evaluated using the Short-Form 36 Health Survey (SF-36) questionnaire, which was given to each group of patients before (T0) and after (T1) the OFS or the placebo treatment. These questions evaluated the possible impact of the OFS on the perceived life quality. The charts show an increasing trend at the end of the treatment (T1) only during the OFS treatment.

**Figure 3:**
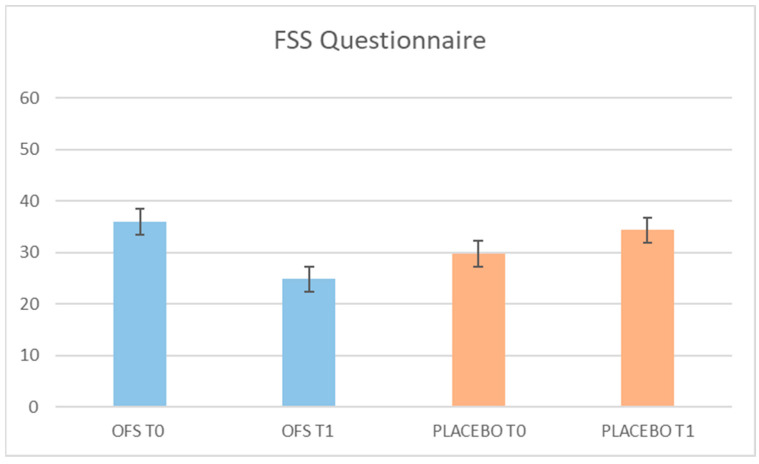
The results of fatigue severity scale (FSS) after oral food supplement and placebo period.

**Figure 4:**
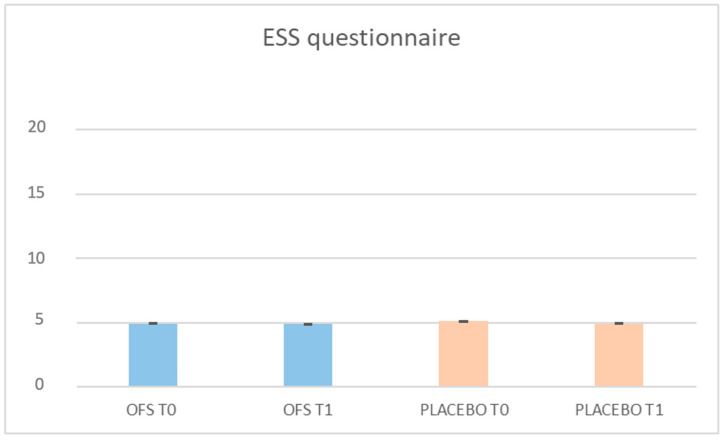
The results of the Epworth Sleepiness Scale (ESS) after the oral food supplement and placebo period.

Figure 3 and Figure 4 show the results of the Fatigue Severity Scale (FSS) and Epworth Sleepiness Scale (ESS) questionnaires, which, respectively, evaluate the degree of fatigue and drowsiness of the enrolled patients. At the end of the study, we observed a statistically significant improvement in fatigue monitored by the FSS questionnaire, exclusively in the period of the OFS treatment (36 ± 6.06 vs. 25 ± 4.13, *p* = 0.0001). On the other hand, after 2 months of OFS treatment, we did not observe any statistically significant difference in the scores of the ESS questionnaire, probably because none of the patients showed a sleep disorder at T0.

Moreover, there were no statistically significant variations in the Prevención con Dieta Mediterránea (PREDIMED) questionnaire, administered during the whole study. This result allows us to overcome the possible bias induced by lifestyle changes on the obtained results.

## 3. Discussion

The clinical presentation of long COVID patients reflects the persistence of a low-grade chronic inflammatory state and it can also have a multisystemic involvement [41]. In fact, long COVID can compromise different organs and impair the patient’s life quality. Furthermore, the systemic inflammatory state can cause CFS onset.

The data obtained from our study showed a statistically significant reduction in some inflammatory biomarkers, such as CRP, NLR and MLR, after the OFS adoption, for 8 weeks. These findings are in line with previous clinical trials conducted on chronic kidney disease patients, in which the same OFS was able to statistically decrease the inflammatory biomarkers, such as CRP and ESR [42].

Therefore, our study confirmed the OFS anti-inflammatory capacity, suggesting a possible synergistic action of NBCs in counteracting the low-grade chronic inflammatory state. This is also corroborated by the observed reduction in NLR and MLR, which are considered emerging inflammatory biomarkers associated with endothelial dysfunction and organ damage [43].

Chronic inflammation characteristic of long COVID, caused by the latent persistence of SARS-CoV-2 in tissues after acute infection [44], may induce a mechanism that promotes systemic hypercoagulability and thrombophilia through the hyperactivation of procoagulant factors and through the formation of thrombotic microclots resistant to fibrinolysis [3]. When SARS-CoV-2 binds to endothelial cells and platelets through angiotensin-converting enzyme 2 (ACE2) and transmembrane serine protease 2 (TMPRSS2) receptors, it promotes endothelial dysfunction and the platelets’ hyperactivation; this bond induces the activation of the coagulation cascade and the formation of thrombotic microclots [3].

The identification of OFSs with anti-inflammatory and antioxidant activity would seem to represent a valid strategy for the treatment and the prevention of long COVID. A previous pilot study showed that an OFS, characterized by anti-inflammatory and antioxidant properties, improves clinical symptoms in long COVID patients [13].

Specifically, the NBCs that make up the OFS and which were investigated in our study are rosehip, zinc and *Echinacea angustifolia*, propolis and royal jelly. *Echinacea angustifolia*, rich in flavonoids, polyphenols, polyols, polysaccharides and glycoproteins with immunostimulating activity, has shown antimicrobial and anti-inflammatory properties. The aforementioned NBCs seem to show a possible impact against SARS-CoV-2, since they would inhibit the Mpro protease, involved in the viral replication [20].

Rosehip is rich in vitamin C and polyphenolic compounds, and therefore it has antioxidant and anti-inflammatory effects. It has been found that the vitamin C content of rosehip by far exceeds that of citrus fruits (30–1300 mg/100 g). The high content of polyphenols and vitamin C would play a key role in cellular detoxification, as both compounds are able to reduce the formation of reactive oxygen species (ROS) [45].

The reduction in inflammatory biomarkers, observed after 8 weeks of OFS treatment, can also be caused by zinc anti-inflammatory action. In our OFS, we assessed the dose of zinc *per* tablet, which was 4.5 mg; therefore, the daily intake of zinc was 9 mg, in line with the maximum tolerable daily dose (25 mg/day) reported in LARN (Reference Nutrient and Energy Intake Levels) [46]. Zinc acts as a modulator of the immune response and its deficiency stimulates inflammation by increasing pro-inflammatory cytokine production [47].

In fact, several studies have shown that zinc levels are inversely related to the concentration of pro-inflammatory cytokines such as IL-6, IL-8 and TNF-α. In addition, zinc deficiency causes an increase in the activation of NF-κB, a transcription factor involved in the expression of many proinflammatory genes. In vitro studies have pointed out that zinc reduces the activation of NF-κB and its target genes, such as TNF-α and IL-1β, and increases the gene expression of A20 and PPAR-α, proteins with anti-inflammatory action [48].

Therefore, the administration of a zinc-based OFS would seem to reduce inflammatory biomarkers, thus supporting the hypothesis of its anti-phlogistic action, as demonstrated in our study [49]. It is also useful to underline the antioxidant power of zinc. The latter, in fact, delays long-term oxidative processes by inducing the expression of metalloproteins, rich in cysteine with a high affinity for heavy metals, which act as powerful electrophilic scavengers and cytoprotective agents. In addition, zinc increases the activation of antioxidant proteins and enzymes, such as glutathione and catalase [48].

Our study also revealed a statistically significant increase in serum values of vitamin D after the OFS assumption. Vitamin D affects the activity of the immune system, modulating the inflammatory process. Several studies have found an inverse correlation between the serum levels of vitamin D and the markers of inflammation [50], confirming the results obtained in our study. Moreover, vitamin D supplementation strengthens the immune response. It is common that patients with chronic inflammatory diseases have low levels of vitamin D [51]. There is also a correlation between vitamin D deficiency and autoimmune diseases, such as multiple sclerosis, diabetes mellitus type 1 and Crohn’s disease [51]. Furthermore, an improvement in vitamin D serum levels appears to be correlated with fatigue reduction. In fact, several studies have highlighted that hypovitaminosis D aggravates chronic tiredness in patients presenting this condition [52]. At the same time, a double-blind placebo-controlled clinical trial underlined the importance of correcting vitamin D levels in order to reduce self-perceived fatigue in patients who showed chronic fatigue [53]. Such evidence is compatible with our results, where the improvement in vitamin D levels after the OFS treatment, in association with the reduction in the above-mentioned inflammatory biomarkers, is linked with the decrease in chronic fatigue symptoms and with the improvement of the quality of life. The first was monitored by the FSS questionnaire and the other one by the SF-36 questionnaire.

The limits of our study are (i) the non-monitoring in all study time-points of the zinc and vitamin C serum levels and (ii) the small sample size.

## 4. Materials and Methods

### 4.1. Analytical Characterization of OFS

The chemical composition of OFS is shown in Table 5.

Before the in vivo study, the OFS was analyzed to determine the amount of total polyphenols and their anti-radical activity (Folin–Ciocalteu method and DPPH-1,1-diphenyl-2-picrylhydrazyl test, respectively). Subsequently, High Performance Liquid Chromatography/Ultraviolet (HPLC/UV) analysis for the quantitative determination of ascorbic acid (vitamin C) was conducted.

#### 4.1.1. Determination of the Quantity of Total Polyphenols: Folin–Ciocalteu Method

The total polyphenol content and the evaluation of the antioxidant activity were carried out through the Folin–Ciocalteu method, described by Singleton et al. (1999) [54]: 0.5 mL of distilled water and 125 µL of Folin–Ciocalteu reagent were added to 125 µL of extract. The mixture was left to react in the dark for 6 min and subsequently 1.25 mL of a 25% sodium bicarbonate solution was added and brought to volume with 1 mL of distilled H_2_O. The mixture was left to stand for 85 min in the dark, and then centrifuged for 5 min at 4500 rpm; the supernatant was then taken and the absorbance at 725 nm was measured. Total polyphenols are expressed in gallic acid equivalents with a calibration curve in gallic acid (Table 6).

#### 4.1.2. Determination of Anti-Radical Activity: DPPH Test

The anti-radical activity of the analyzed samples, which can be correlated with the antioxidant activity, was evaluated using the DPPH• (1,1-diphenyl-2-picrylhydrazyl radical) test. The procedure described by Gulcin and Alwasel (2023) [55] was performed, with minor modifications. A radical solution, prepared with 2.5 mg of DPPH in 100 mL of EtOH, was used. The kinetics of the extract were determined and the sample was analyzed to evaluate the reduction in the reagent-oxidizing activity. The analysis was carried out on the sample of the extract obtained previously and further diluted 1:10 in 70:30 ETOH/H_2_O pH 3.2 for HCOOH. The reaction kinetics were obtained by reading the relative absorbance at 517 nm of the solution sample with the radical at set times. The anti-radical activity is reported in Table 7.

#### 4.1.3. HPLC/UV Chromatographic Analyses

One capsule (400 mg) of OFS was extracted with 4 mL of H_2_O pH = 2.4 for HCOOH, for 30 min in the dark. The sample was then centrifuged and subjected to chromatographic analysis HPLC-UV for the evaluation of vitamin C content. The quantitative analysis was obtained using an HP-1260 liquid chromatograph (Agilent Technologies, Palo Alto, CA, USA), and for the chromatographic separation a column (Interchim C18 250 × 4.6 mm i.d. 5µm) was used. The analysis was carried out using H_2_O (pH 3.2 for HCOOH) as the mobile phase and CH_3_CN in the isocratic phase: 95% H_2_O in 8 min, flow rate 0.8 mL/min. The quantitative analysis was conducted with the aid of a 4-point acid calibration curve ascorbic (r^2^ ≥ 0.998) at 280 nm. The OFS showed a vitamin C content of 22.17 mg/g (8.87 mg/cp).

### 4.2. Enrolled Patients

The study population included 33 patients, 14 males and 19 females, with a mean age of 47.6 ± 16.05 years, who picked up SARS-CoV-2 infection from 1 to 6 months before the start of the study (Table 8).

The patients were enrolled at the Department of Medical Sciences of Policlinico Tor Vergata (PTV) of Rome following the protocol code RS 150.21 (approved on 4 November 2021) by Independent Ethical Committee of PTV. Before signing their informed consent, all patients were properly informed about the study characteristics. The recommended dosage of OFS or placebo was 2 tablets per day, for 2 months of treatment (OFS or placebo). OFS and placebo were identical in their appearance and in the taste and were supplied in non-distinctive white boxes. All enrolled patients underwent a vaccination, as highly recommended by the Italian Ministry of Health.

The inclusion criteria were:

- Both sexes;
- Age between 18 and 75 years;
- Body mass index (BMI) between 18.5 and 25 kg/m^2^;
- Patients with previous COVID-19 infection (ascertained by double-positive nose-pharyngeal molecular swab) in remission from 1 to 6 months (as confirmed by the negativity of the molecular nose-pharyngeal swab for SARS-CoV-2).

The exclusion criteria were:

- Medications;
- Pregnancy and lactation;
- Both solid and hematological active phase neoplasms;
- HIV, HbsAg and HCV-positive patients;
- Not accepting informed consent;
- Any chronic pathological condition;
- Possible allergies to OFS components;
- Assumption of other OFSs with anti-inflammatory and antioxidant properties in the previous two months.

**Figure 5:**
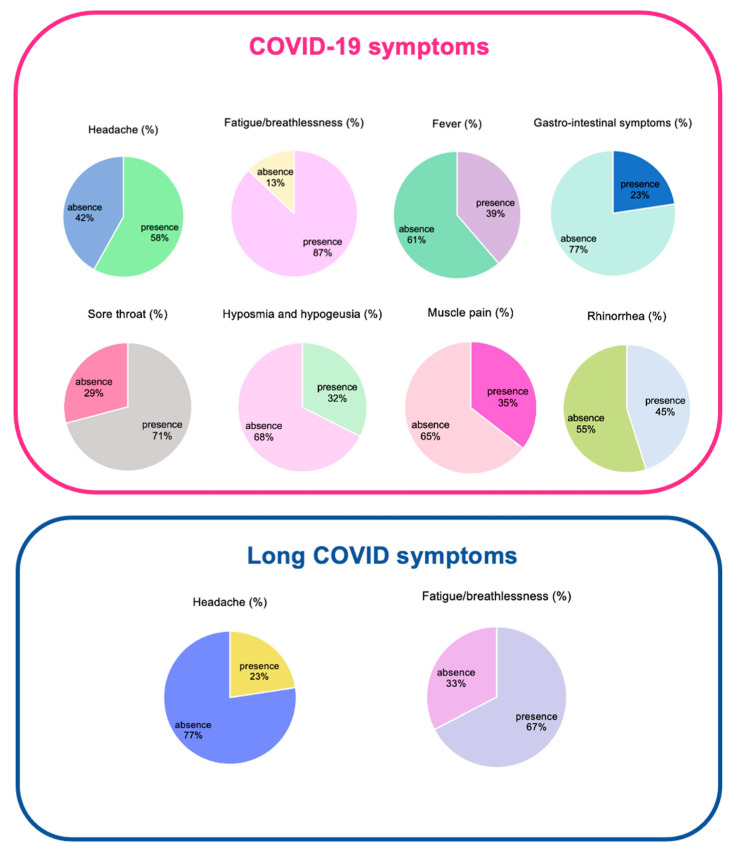
Main symptoms shown by the enrolled patients during COVID-19 infection and long COVID syndrome.

In Figure 5, we report the main symptoms of our study population. Patients were asymptomatic or they showed mild symptoms of COVID-19 infection.

The study population was divided into two homogeneous groups (group A and group B) for age, sex and BMI.

**Figure 6:**
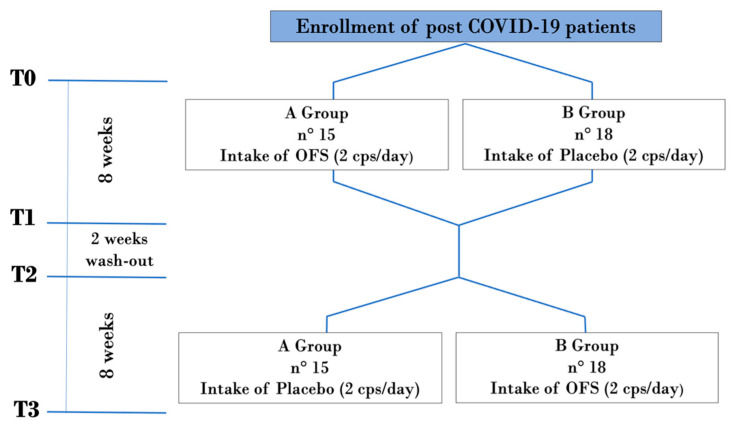
Flow-chart of double-blind placebo-controlled randomized trial. Abbreviations: T: Time point of the study.

The study was structured in 4 time-points (Figure 6): T0, enrollment time; T1, after 8 weeks from the start of the study, namely, after the first two months of treatment with the OFS or with the placebo; T2, after the wash-out period of two weeks, namely, after ten weeks from the beginning of the study; and T3, after a further two months’ time from T2, namely, at the end of the 18th week from the beginning of the study.

### 4.3. Laboratory Parameters

A code was assigned to all blood samples collected to make them anonymous. At the end of the study, all the samples collected were destroyed.

At the enrollment (T0), all patients were subjected to an accurate pathological medical history, in which information regarding the daily pharmacological therapy taken on by patients was also collected.

At different time-points of the study (T0, T1, T2, T3), laboratory tests were carried out by collecting a peripheral venous blood sample and a urine sample. In detail, the following bloodwork and urinary analyses were performed: complete blood count and calculation of inflammatory indices (NLR, PLR, MLR and LMR), serum creatinine, estimated glomerular filtration rate (eGFR), azotemia, inflammatory parameters such as ESR and CRP, vitamin D, serum anti-SARS-CoV-2 antibody level, complete urine test and albuminuria from a morning urine sample. 

The collected sera were centrifugated for 10 min at 2000× *g* and then processed as fresh samples. The white blood cell (WBC) count was measured by an automated hematological analyzer, using EDTA blood tubes (BC-6800 plus, Mindray, Shenzen, China). Serum levels of creatininemia, azotemia, CRP and vitamin D were determined using an Abbott Alinity assay (Abbott Diagnostics, Chicago, IL, USA). Serum levels of anti-SARS-CoV-2 antibody were measured using a CL-series SARS-CoV-2 IgG assay (CL-1200, Mindray, Shenzen, China). The ESR test was performed on blood samples collected in EDTA blood tubes; the analyzer could measure ESR directly from the capped EDTA tube for an executive period of 20 min of optical reading. Urine samples were analyzed with the Sysmex UF-1000i system, while albuminuria was determined using an Abbott Alinity assay (Abbott Diagnostics, Chicago, IL, USA).

### 4.4. Blood Pressure Monitoring

Systolic and diastolic blood pressure (BP) were monitored during the whole study. The measurements were recorded by an automatic sphygmomanometer: the arm cuff was located at the heart level and the BP was measured three times and the third measurement was recorded. The heart rate was also measured [56].

### 4.5. Body Composition Analysis

In all patients, we evaluated anthropometric parameters such as body weight (kg) and height (m), according to standard methods [57]. The BMI was calculated as body weight divided by height squared (kg/m^2^).

Patients performed a body composition assessment using bioimpedance analysis (BIA) measured through a BIA EFG Plus (ESTOR spa–Milan, Italy). Through the BIA, total body water (TBW), intracellular water (ICW), extracellular water (ECW), body cell mass index (BCMI), fat-free mass (FFM), fat mass (FM) and muscle mass (MM) were evaluated [58].

### 4.6. Questionnaires

For the evaluation of life quality perception, the SF-36 questionnaire was administered to all enrolled patients. The SF-36 questionnaire, in self-administration mode, is very useful for evaluating the patient’s health-related quality of life. It consists of 36 questions divided into 9 spheres: the perception of general health (5 questions), health changes (1 question), physical functioning (10 questions), perception of pain (2 questions), social functioning (2 questions), emotional well-being (5 questions), fatigue (4 questions), activity limitations due to health status (4 questions) and activity limitations due to emotional state (3 questions). For each sphere, it is possible to obtain values between 0 and 100 which directly correlate with psychophysical well-being [59].

To evaluate the fatigue and the drowsiness of the enrolled patients, the FSS and the ESS questionnaires were administered, respectively.

The FSS is a questionnaire designed to assess the severity of fatigue, based on its impact on the patient’s daily activities. This questionnaire has also been recently validated in post-COVID patients, both hospitalized and non-hospitalized. It includes 9 questions with a minimum score of 9 (not fatigued) to a maximum score of 63 (very fatigued) [60].

The ESS is widely used and validated as a drowsiness measurement tool, and can negatively impact the patient’s quality of life. It is based on the evaluation, self-reported by the patient, of the probability of falling asleep in different situations that usually do not stimulate sleep. This questionnaire includes 8 questions, with a minimum score of 0 (the patient does not have any sleep disorder) to a maximum score of 24 (the patient suffers from a serious sleep disorder) [61].

Moreover, in order to exclude the influence of any lifestyle changes on the parameters examined, and to evaluate the real effects of the OFS, the PREDIMED questionnaire, which measures the adherence to the Mediterranean diet, was administered during the study [62].

### 4.7. Statistical Analysis

The statistical analysis was performed using the Windows Social Science Statistical Package, version 15.0 (SPSS, Chicago, IL, USA). Descriptive statistics consisted of the standard deviation (SD) for the parameter with normal distribution (after confirmation with histograms and the Kolmogorov–Smirnov test) and the median and interval (min; max) for variables with non-normal distribution. The test to verify the homogeneity of the anagraphic–anthropometric data among the patients enrolled in the study was conducted with one-way ANOVA.

The Chi-squared test was carried out for the execution of the inferential statistical analysis and the evaluation of the possible associations or dependencies between two categorical variables. A *p* value ≤ 0.05 was considered statistically significant.

## 5. Conclusions

**Figure 7:**
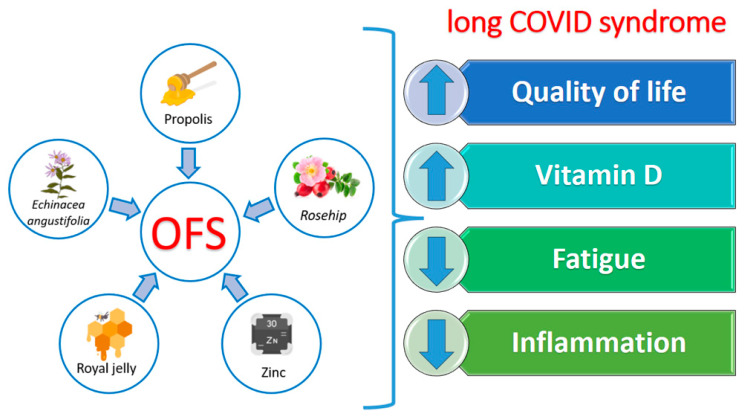
The synergistic effect of ingredients contained in the oral food supplement in long COVID syndrome. Abbreviation: OFS, oral food supplement.

This double-blind, placebo-controlled randomized trial would seem to confirm the beneficial role of the OFS in long COVID patients. This hypothesis is supported by our results, which allowed us to observe a positive impact of OFS treatment on the inflammatory state and, at the same time, an improvement in life quality and fatigue perception, as evidenced by the positive trend obtained by SF-36 and FSS questionnaires. Therefore, the OFS would seem to be useful in the treatment of the chronic inflammatory state, typical of long COVID patients. In fact, thanks to the synergistic effect of NBCs contained in the tested OFSs, we observed its ability to counteracting the inflammatory state, with a consequent reduction in patients’ chronic fatigue and a noticeable improvement in their quality of life (Figure 7).

## Figures and Tables

**Table 1:** Laboratory parameters monitored before (T0) and after (T1) treatment with the OFS and before (T0) and after (T1) the placebo.

Parameters	OFS		PLACEBO		*p*
	T0	T1	T0	T1	
Red blood cells (millions/μL)	4.60 ± 0.41	4.73 ± 0.53	4.72 ± 0.53	4.61 ± 0.41	n.s.
Hemoglobin (g/dL)	13.94 ± 1.50	14.06 ± 1.50	14.03 ± 1.53	14.07 ± 1.51	n.s.
White blood cells (thousands/μL)	6.43 ± 1.54	6.50 ± 1.38	6.17 ± 1.52	6.29 ± 1.51	n.s.
Platelets (thousands/μL)	230.81 ± 56.56	231.19 ± 53.34	236.13 ± 47.58	234.29 ± 48.46	n.s.
Neutrophils (thousands/μL)	3.74 ± 1.29	3.74 ± 1.14	3.54 ± 1.19	3.50 ± 0.98	n.s.
Lymphocytes (thousands/µL)	2.06 ± 0.61	2.12 ± 0.72	2.02 ± 0.56	2.19 ± 0.06	n.s.
Monocytes (thousands/μL)	0.50 ± 0.18	0.48 ± 0.18	0.47 ± 0.14	0.45 ± 0.13	n.s.
Eosinophils(thousands/μL)	0.12 ± 0.10	0.13 ± 0.13	0.11 ± 0.10	0.12 ± 0.11	n.s.
Creatininemia (mg/L)	0.73 ± 0.16	0.76 ± 0.17	0.76 ± 0.18	0.76 ± 0.17	n.s.
eGFR (mL/min/1.73 m^2^)	103.49 ± 13.55	100.48 ± 15.27	100.61 ± 14.94	100.92 ± 15.81	n.s.
Azotemia (mg/dL)	32.71 ± 7.84	33.19 ± 9.17	32.68 ± 8.43	33.48 ± 10.43	n.s.
Vitamin D (mg/dL)	27.96 ± 10.25	34.54 ± 12.53	31.20 ± 10.65	33.98 ± 13.40	0.0005
D-dimer (ng/mL)	350.4 ± 80	335.9 ± 49	360.7 ± 70	350.1 ± 75	n.s.
Ferritin (ng/mL)	200.7 ± 80.3	190.3 ± 70.9	193.2 ± 90.1	210.1 ± 60.8	n.s.
Serum anti-SARS-CoV-2 antibody (U/mL)	10,304.73 ± 10,795.05	12,837.32 ± 15,230.70	10,466 ± 10,653.86	9762.73 ± 10,771.67	n.s.
Albuminuria on urine sample morning (mg/dL)	20.01 ± 51.53	25.96 ± 47.84	23.14 ± 43.11	21.27 ± 52.97	n.s.

Data expressed as mean ± standard deviation; applied test: *t*-test for paired data. Values of *p* ≤ 0.05 are considered statistically significant. Abbreviations: eGFR: estimated glomerular filtration rate; n.s.: not significant; OFS: oral food supplement.

**Table 2:** Inflammatory parameters monitored before (T0) and after (T1) the OFS treatment and before (T0) and after (T1) the placebo adoption.

Parameters	OFS		PLACEBO		*p*
	T0	T1	T0	T1	
CRP (mg/L)	2.77 ± 2.5	1.44 ± 1.70	2.15 ± 2.40	1.90 ± 2.52	0.0145
ESR (mm/h)	14.25 ± 13.16	11.35 ± 10.93	11.39 ± 9.61	15.55 ± 12.81	n.s.
NLR	2.08 ± 0.81	1.77 ± 1.03	1.85 ± 0.66	1.70 ± 0.61	0.0455
PLR	119.75 ± 40.27	121.56 ± 49.86	121.91 ± 21.5	116.31 ± 39.21	n.s.
MLR	0.26 ± 0.14	0.13 ± 0.05	0.25 ± 0.08	0.21 ± 0.06	0.0005
LMR	4.54 ± 1.43	4.63 ± 1.39	4.42 ± 1.30	5.01 ± 1.20	n.s.

Data expressed as mean ± standard deviation; applied test: *t*-test for paired data. Values of *p* ≤ 0.05 are considered statistically significant. Abbreviations: CRP: C-reactive protein; ESR: erythrocyte sedimentation rate; MLR: monocyte/lymphocyte ratio; LMR: lymphocyte/monocyte ratio: n.s.: not statistically significant; NLR: neutrophil/lymphocyte ratio; OFS: oral food supplement; PLR: platelet/lymphocyte ratio.

**Table 3:** Parameters related to the body composition of patients monitored before (T0) and after (T1) the OFS treatment and before (T0) and after (T1) the placebo adoption.

Parameters	OFS		PLACEBO		*p*
	T0	T1	T0	T1	
Weight (kg)	68.25 ± 10.77	67.52 ± 10.70	67.29 ± 10.85	67.80 ± 11.10	n.s.
Height (m)	1.67 ± 0.09	1.67 ± 0.09	1.67 ± 0.09	1.67 ± 0.09	n.s.
BMI (kg/m^2^)	24.41 ± 3.28	24.15 ± 3.21	24.19 ± 3.43	30.68 ± 35.91	n.s.
RZ (Ohm)	52.94 ± 10.76	53.16 ± 9.81	53.64 ± 9.31	54.3 ± 9.89	n.s.
RX (Ohm)	541.03 ± 109.59	535.74 ± 93.10	545.10 ± 92.18	511 ± 123	n.s.
PA (°)	5.67 ± 1.00	5.76 ± 0.89	6.1 ± 2.63	6.02 ± 0.81	n.s.
TBW%	55.60 ± 6.00	55.91 ± 5.78	55.5 ± 5.15	57.3 ± 6.19	n.s.
ICW%	52.25 ± 5.20	52.75 ± 4.73	52.41 ± 3.88	54.24 ± 3.66	n.s.
ECW%	47.75 ± 5.20	47.25 ± 4.73	47.59 ± 3.87	45.76 ± 3.66	n.s.
FM%	24.69 ± 7.83	23.69 ± 8.14	24.41 ± 7.09	22.03 ± 8.29	n.s.
FFM%	75.30 ± 7.84	76.31 ± 8.14	75.59 ± 7.09	77.97 ± 8.29	n.s.
BCM%	51.61 ± 5.57	52.12 ± 5.07	51.51 ± 4.02	53.62 ± 4.10	n.s.
BCMI (kg/m^2^)	9.54 ± 2.28	9.63 ± 2.02	9.45 ± 1.74	10.12 ± 1.90	n.s.

Data expressed as mean ± standard deviation; applied test: *t*-test for paired data. Values of *p* ≤ 0.05 are considered statistically significant. Abbreviations: BCM: Body Cell Mass; BCMI: Body Cell Mass Index; BMI: Body Mass Index; ECW: Extracellular Water; FFM: Fat-Free Mass; FM: Fat Mass; ICW: Intracellular Water; n.s.: Not Significant; PA: Phase Angle; RX: Reactance; RZ: Resistance; TBW: Total Body Water.

**Table 4:** Blood pressure parameters and heart rate monitored before (T0) and after (T1) the OFS treatment and before (T0) and after (T1) the placebo assumption.

Parameters	OFS		PLACEBO		*p*
	T0	T1	T0	T1	
Systolic blood pressure (mmHg)	120.26 ± 14.01	114.97 ± 12.45	119 ± 15.67	116.19 ± 13.32	n.s.
Diastolic blood pressure (mmHg)	73.16 ± 8.07	71.52 ± 7.96	73.87 ± 9.13	73.19 ± 9.02	n.s.
Heart rate (bpm)	67.90 ± 9.96	68.32 ± 9.14	69.29 ± 11.61	66.74 ± 10.30	n.s.

Data expressed as mean ± standard deviation; applied test: *t*-test for paired data. Values of *p* ≤ 0.05 are considered statistically significant. Abbreviations: n.s.: not significant, OFS: oral food supplement.

**Table 5:** Composition of the oral food supplement according to LARN. % VNR = percentage of the reference nutritional value.

Components	For Daily Dose (2 cp)	%VNR/2 cp
Zinc	9 mg	90%
*Echinacea angustifolia* e.s. tit. min. 4% echinacoside	30 mg	-
Rosehip e.s. tit. min. 10% Vitamin C	20 mg 14 mg	17.5%
90%—Propolis powder cryo macinate tit. min. 12.15% in galangin total flavonoids	5.56 mg	-
Lyophilized royal jelly	40 mg	-

**Table 6:** Total polyphenols for the capsule of oral food supplement, expressed as gallic acid (mg).

Sample	Total Polyphenols for Capsule, Expressed as Gallic Acid (mg)
OFS	43.98

**Table 7:** Anti-radical activity of oral food supplement.

Sample Dilution	Anti-Radical Activity%
1:10	92.15

**Table 8:** Study population features.

Total patients (n)	33
Sex (male/female)	14/19
Mean age (years)	47.6 ± 16.05
Time since infection (days)	73.7 ± 35.9
Duration of SARS-CoV-2 infection (days)	12.3 ± 5.7

## Data Availability

Data is contained within the article.
